# Alanine Substitution to Determine the Effect of LR5 and YR6 Rice Peptide Structure on Antioxidant and Anti-Inflammatory Activity

**DOI:** 10.3390/nu15102373

**Published:** 2023-05-18

**Authors:** Yun-Hui Cheng, Bu-Qing Liu, Bo Cui, Li Wen, Zhou Xu, Mao-Long Chen, Hao Wu

**Affiliations:** 1 School of Food Science and Engineering, Qilu University of Technology (Shandong Academy of Sciences), Jinan 250353, China; 2 School of Food Science and Bioengineering, Changsha University of Science & Technology, Changsha 410114, China

**Keywords:** bioactive peptide, amino acid, hydrophobic, anti-inflammatory, antioxidant

## Abstract

The relationship between the structure of peptides LR5 (LHKFR) and YR6 (YGLYPR) and their antioxidant and anti-inflammatory activity remains unclear. Herein, leucine, tyrosine, proline, and phenylalanine at different positions in the peptides were replaced by Alanine (Ala), and two new pentapeptides (AR5 and LAR5) and four hexapeptides (AGR6, YAR6, YLR6, and YGR6) were obtained. The effect of Ala replacement on the hydrophobicity, cytotoxicity, NO inhibition rate, and active oxygen radical scavenging ability of these peptides and their antioxidant and anti-inflammatory abilities were investigated. The results indicated that the hydrophobicity of the peptides was associated with their amino acid composition and their specific sequence. However, hydrophobicity had no significant effect on cytotoxicity. Ala replacement was shown to enhance hydrophobicity and consequently increased the antioxidant and anti-inflammatory activity of the peptides. The molecular docking studies indicated that the amino acid interactions of the peptide with the Keap1 protein influenced the hydrophobicity and thus affected the antioxidant activity of the peptide.

## 1. Introduction

Rice protein is recognized as a high-quality plant protein among cereal proteins because it does not contain anti-nutritional factors and allergens and will therefore not induce allergic reactions [1]. However, the content of insoluble glutelin in rice protein is very high (80–90%), and there are a large number of disulfide bonds between molecules, making it less soluble [2,3]. Recently, the development and commercial utilization of rice protein has attracted increasing attention. Because of its favorable characteristics, several rice protein products have been developed for various uses, including its use as a food additive to improve physicochemical properties, as a nutritional protein powder with more than 80% protein content, and even its use to generate bioactive peptides with special functions [4].

Bioactive peptides can be released from parent proteins in vivo or in vitro by enzymolysis, and they can perform specific physiological functions. Rice active peptide is a mixture of rice proteins obtained by enzymatic hydrolysis, including polypeptides with different molecular weights, proteins without enzymatic hydrolysis, free amino acids, and inorganic salts. Many bioactive rice peptides have been reported, including those with antioxidant [5], antihypertensive [6], and anti-inflammatory activities [7]. In general, antioxidant peptides can effectively eliminate excess reactive oxygen species in the body, thus protecting the normal structure and function of cells and mitochondria and preventing the occurrence of lipid peroxidation, helping the human body to resist disease [8]. Anti-inflammatory peptides can enhance the immune ability of the human body, reinforce the phagocytosis of macrophages, and improve immune function against external pathogens [9]. Therefore, research on antioxidant and anti-inflammatory peptides has become a hot topic in the field of bioactive peptides.

So far, researchers have isolated a variety of peptides with anti-inflammatory activities from milk protein, Baijiu vinasse, rice protein, collagen protein [10], and scallop by-products [11]. For example, Chen and colleagues separated antioxidant and anti-inflammatory collagen peptides from milkfish scale (MSCP) using the pepsin-soluble collagen method [12], and Peng and colleagues also separated bioactive peptides with antioxidant and anti-inflammatory properties from Baijiu vinasse [13]. However, these studies have rarely explored the relationship between the structure of peptides and their antioxidant and anti-inflammatory activity. In fact, the peptide amino acid sequences are closely related to their function [14]. Therefore, the structural modification of peptides could be an effective method to alter and strengthen desirable functions, which could be achieved through the replacement of amino acid residues [15], gene recombination [16], or the addition of positive charges [17]. Yang and colleagues found that peptides rich in arginine (Arg) exhibited broad-spectrum bactericidal activity against pathogenic bacteria [18]. Another study also indicated that introducing positively charged amino acids into peptide chains could increase peptide antimicrobial activity [19]. The structure–activity relationship of antimicrobial peptides has also been studied by amino acid substitution [5]. Because alanine (Ala) has a neutral, short side chain and no charge, many studies have used Ala replacement to determine the key amino acids that produce functional active peptides. For example, Pei and colleagues identified key residues in the PT5 peptide and determined a core sequence for PT5 binding to p38α by substituting threonine (Thr) and aspartic acid (Asp) with Ala [20].

Pentapeptide LR5 (LHKFR) and hexapeptide YR6 (YGLYPR) were isolated and purified from the rice protein from a previous study and were shown to have antioxidant and anti-inflammatory activity. However, the relationship between the structure of those peptides and their antioxidant and anti-inflammatory activities remains unclear. Consequently, in this study, two new pentapeptides, AR5 (the detailed sequence is AHKFR) and LAR5 (LHKAR), and four new hexapeptides, AGR6 (AGLYPR), YAR6 (YGAYPR), YLR6 (YGLAPR), and YGR6 (YGLYAR), were obtained by replacing leucine (Leu), phenylalanine (Phe), tyrosine (Tyr), and proline (Pro) with alanine (Ala) in the original peptides LR5 and YR6 to study the relationship between peptide structure and activity. The effect of these peptides on the proliferation and anti-inflammatory activity of mouse macrophage cells induced by lipopolysaccharides (LPS) was investigated using the MTT method. The scavenging capacity of superoxide anion radicals (O_2_^−^**·**) and hydroxyl radicals (HO**·**) was also investigated using phloroglucinol luminol and phenanthroline chemiluminescence, respectively. The cytotoxicity, anti-inflammatory activity, and antioxidant activity of the original and replacement peptides were studied to comprehensively evaluate the effect of amino acid replacement on antioxidant and anti-inflammatory activities. Furthermore, the molecular docking model and molecular dynamics simulation were used to study the relationship between the rice peptide and the replacement peptide structure on the antioxidant and anti-inflammatory activity at the molecular level.

## 2. Materials and Methods

### 2.1. Material

LPS, glutathione (GSH), phloroglucinol, and luminol were purchased from Sigma-Aldrich Co., Ltd. (St. Louis, MO, USA). Dulbecco’s modified Eagle’s medium (DMEM)-high glucose, used for cell growth, was purchased from Thermo Fisher Scientific Co., Ltd. (Waltham, MA, USA). Mouse macrophages (RAW264.7) were purchased from the BeNa Culture Collection (Beijing, China). All other reagents were purchased from Sinopharm Chemical Reagent Co., Ltd. (Shanghai, China). The original and replacement peptides LR5 (LHKFR), AR5 (AHKFR), LAR5 (LHKAR), YR6 (YGLYPR), AGR6 (AGLYPR), YAR6 (YGAYPR), YLR6 (YGLAPR), and YGR6 (YGLYAR) were synthesized by GL Biochem Co., Ltd. (Shanghai, China) with a purity of >95%. The structure of all of these peptides is shown in Figure S1. The detailed amino acid sequences of these peptides are presented in Table S1.

### 2.2. Determination of the Hydrophobicity of Original and Replacement Rice Peptides

The hydrophobicity of LR5, AR5, LAR5, YR6, AGR6, YAR6, YLR6, and YGR6 was measured using reversed-phase high-performance liquid chromatography equipment (RP-HPLC, Waters e2695; Milford, MA, USA) which was equipped with a UV detector and a Boston Green ODS-AQ column (250 × 4.6 mm, 5 μm) equilibrated with mobile phase solvent A (100% [*v*/*v*] water and 0.1% trifluoroacetic acid) and solvent B (100% [*v*/*v*] acetonitrile and 0.1% trifluoroacetic acid) at 25 °C and 220 nm. A 10 μL aliquot filtered by Millipore filtration (through a 0.45 μm filter) was injected into the RP-HPLC system and the flow rate was adjusted to 1.0 mL/min.

### 2.3. Determination of the Superoxide Anion Radicals (O_2_^−^**·**) Scavenging Ability of the Original and Replacement Rice Peptides

The superoxide anion radical (O_2_^−^**·**) scavenging ability of each peptide was determined using the previously published pyrogallol–biochemiluminescence method [7]. The detailed steps of this protocol were as follows: 100 μL of sample and 100 μL of pyrogallol (1 mM) were added into 800 μL of luminol–carbonate buffer solution system (pH 10.2, 0.1 mM). Then, the mixed sample was put into the reaction chamber and measured using a bioluminescence instrument to record a luminescence kinetics curve. The O_2_^−^**·** scavenging efficiencies of different peptides were calculated according to Formula (1), based on the luminous integral intensity at the peak. The half maximal inhibitory concentration (IC_50_) value was used to evaluate the O_2_^−^**·** scavenging ability of the peptide in the sample. GSH was used as a control sample.

(1) $${O_{2}}^{-}\cdot \;\mathrm{scavenging}\;\mathrm{efficiency}\;(\%)=\frac{A_{0}-A_{1}}{A_{0}}\;\times \;100\%$$

where A_0_ was the luminous intensity of the blank group (in which deionized water was added), and A_1_ was the luminous intensity of the experimental group (in which peptide was added).

### 2.4. Determination of the Hydroxyl Radical (HO**·**)Scavenging Ability of the Original and Replacement Rice Peptides

The hydroxyl radical (HO**·**) scavenging ability of each peptide was determined using the previously published 1,10-phenanthroline-biochemiluminescence method [7]. In total, 50 μL of each sample (peptide), 50 μL of 1,10-phenanthroline (1.5 mM), 50 μL of CuSO_4_ solution (1.25 mM), 20 μL of L-ascorbic acid (0.25 mM), and 50 μL of H_2_O_2_ (*v*/*v*, 25%) were added into 780 μL of borax–borate solution (pH 7.5). Then, the mixed sample was put into the reaction chamber and measured using a bioluminescence instrument to record luminescence kinetics curves. The HO**·** scavenging efficiency of the different peptides was calculated according to Formula (2). The IC_50_ value was used to evaluate the HO**·** scavenging ability in the sample. GSH was used as a control sample.

(2) $$\mathrm{HO}\cdot \;\mathrm{scavenging}\;\mathrm{efficiency}\;(\%)=\frac{A_{0}-A_{1}}{A_{0}}\;\times \;100\%$$

where A_0_ indicates the luminous intensity of the blank group (no added hydrogen peroxide) and A_1_ indicates the luminous intensity of the experimental group (with hydrogen peroxide added).

### 2.5. Effects of the Original and Replacement Peptides on Proliferation and Toxicity in RAW264.7 Cells

The MTT colorimetry method was used to determine the effects of original rice peptides and replacement peptides on RAW264.7 cell proliferation, and the toxic effects of these peptides on the cells [21]. RAW264.7 cells were cultured in DMEM high glucose medium containing 10% (*v*/*v*) fetal bovine serum (FBS) and 1% (*v*/*v*) of penicillin–streptomycin solution (10,000 U/mL of penicillin and 10,000 U/mL of streptomycin). The cells were cultured at 37 °C in a humidified incubator (Boxun Co., Ltd., Shanghai, China) with 5% (*v*/*v*) CO_2_, with sub-culturing every three days.

Briefly, pre-cultured, logarithmic phase cells were inoculated into 96-well plates (with six double wells for each group), at a cell density of 6.0 × 10^4^ cells/mL. Then, 200 μL of cell suspension was added into the wells and incubated for 6 h to make the cells adhere to the well wall. After that, different concentrations of samples (12.5, 25, 50, and 100 μg/mL) were added to four of the six corresponding wells for each group. Sterile water and immunoglobulin G (IgG) were used as the blank and positive control groups, respectively. The samples were subsequently incubated in a humidified incubator with 5% (*v*/*v*) CO_2_ at 37 °C for 24 h, and then 20 μL of MTT solution (5 mg/mL) was added to each well and incubated for a further 4 h. Next, the culture medium supernatant was gently removed from the wells, and 150 μL of dimethyl sulfoxide (DMSO) solution was added into the wells, which were shaken for 10 min at room temperature to ensure that the crystal violet was fully dissolved. Then, the solution was immediately detected at 490 nm. The cytotoxicity was evaluated based on the following stimulating index (SI) value Formula (3):

(3) $$\mathrm{SI}=\frac{{OD}_{1}}{{OD}_{0}}$$

where OD_1_ is the absorbance value at 490 nm of the sample group or the IgG group, and OD_0_ is the absorbance value at 490 nm of the blank group.

### 2.6. Determination of the Anti-Inflammatory Activity of the Original and Replacement Rice Peptides in RAW264.7 Cells

#### 2.6.1. Effect of LPS on Proliferation and Nitric Oxide (NO) Release in RAW264.7 Cells

RAW264.7 cells were cultured according to Section 2.5, except that the cell density was 2.0 × 10^5^ cells/mL. Different concentrations of LPS (0, 0.1, 1, 10, and 100 μg/mL) were added into the wells containing adherent RAW264.7 cells. The samples were subsequently incubated in a humidified incubator with 5% (*v*/*v*) CO_2_ at 37 °C for 24 h. The effect of LPS on the proliferation of RAW264.7 cells was determined using the MTT method.

The amount of NO released by the RAW 264.7 cells was measured using the Griess assay. After the cells were treated with different concentrations of LPS for 24 h, the culture medium in each well was collected and transferred into a new 1.5 mL EP tube by centrifugation at 4 °C and 12,000× *g* for 5 min to remove cell debris. Then, 100 μL of the centrifuged supernatant from each well was added to a single well of a new 96-well plate, and 50 μL of Griess A was added to each well and incubated at 37 °C for 10 min. Then, 50 μL of Griess B was added and incubated for another 10 min. Finally, absorbance values of the solutions in each well were detected at 540 nm using a microplate reader.

#### 2.6.2. Effects of Peptide Pre-Treatment with Different Concentrations of LPS on the NO Released from the RAW264.7 Cells

The RAW264.7 cells were cultured according to Section 2.5. Then, different concentrations of peptides (12.5, 25, 50, and 100 μg/mL) were added to four of the six wells for each peptide. Sterile water and IgG were used as the blank and positive control groups, respectively. After incubation for 12 h, a final concentration of 10 μg/mL LPS was added and incubated for another 24 h. The content of NO was determined using the Griess assay. The inhibition rate of different concentrations of peptides on NO release from the RAW264.7 cells was calculated using the following Formula (4):

(4) $$\mathrm{inhibition}\;\mathrm{rate}\;(\%)=\frac{C_{0}-C_{1}}{C_{0}}\;\times \;100\%$$

where C_0_ is the nitrite concentration of the blank control group and C_1_ is the nitrite concentration of the sample group or the IgG group.

### 2.7. Molecular Docking of Peptides with the Keap1 Protein

Antioxidant analysis at the molecular level was performed by modeling the molecular docking of each peptide with the Keap1 protein. The structure of the peptides was constructed using ChemBioDraw Ultra v14.0. Before the docking analysis, all water and ligand molecules were removed from the crystal structure of the Keap1 (PDB: 2FLU) protein. AutoDock Vina molecular simulation software was used to perform semi-flexible docking between different bioactive peptides and the Keap1 protein. The docking position of the canonical ligand Nrf2 within the Keap1 receptor protein was considered the active center of the docking box to generate a series of text files using Vina Config. The composite structure with the highest affinity (lowest energy) was selected for subsequent analysis, based on the default scoring function of AutoDock Vina. The interaction between the ligands and the receptor protein was analyzed by Ligplot v.2.2.5 software.

### 2.8. Molecular Dynamics Simulation of the Peptides with the Keap1 Protein

Molecular dynamics simulation of the peptides with the keap1 protein was performed using Gromacs 2022.1 software under 298.15 K according to the reference [22] with slight modification. Amber14SB force field was used to calculate the keap1 protein and peptides. Molecular dynamics simulation of 30 ns was conducted on the system, and conformation was saved every 10 ps.

### 2.9. Statistical Analyses

All experimental data were expressed in the form of mean ± standard deviation (SD). Three replicates were performed for each set of experiments. The data were statistically analyzed using SPSS v18.0 software. The results were considered significant at a threshold of *p* < 0.05 and extremely significant at a threshold of *p* < 0.01.

## 3. Results

### 3.1. Analysis of the Hydrophobicity of the Original and Replacement Rice Peptides

The degree of amino acid hydrophobicity can usually be characterized using the value of the hydrophilic index (HI). A higher value of the HI indicates lower hydrophobicity. The HI value of 20 amino acids is shown in Table S2. The hydrophobicity of the original rice peptides (LR5 and YR6) and the replacement peptides (LAR5, AR5, YGR6, AGR6, YLR6, and YAR6) was compared to the corresponding retention time results from RP-HPLC, because of the different strengths of the hydrophobic interaction between the peptide and the column matrix. The hydrophobicity of the newly generated pentapeptides AR5 and LAR5, in which Ala (HI = 1.8) replaced Phe (HI = 2.8) and Leu (HI = 3.8), respectively, had lower hydrophobicity than the original peptide LR5 (Table S3 and Figure S2). When Ala replaced Tyr (HI = −1.3), the hydrophobicity of the new hexapeptide, AGR6, was higher than that of the original peptide (YR6) as expected; however, the hydrophobicity of YLR6 was unexpectedly lower than that of YR6. Further study found that the arrangement of Pro and Arg in YLR6 was altered. The amino acids connected to the N-terminus of Pro in AGR6 and YLR6 were Tyr and Ala, respectively. Since the peptide chain would form a corner at the Pro residue, the difference in amino acids may lead to the formation of steric hindrance between Ala and Pro at the corner of the YLR6 peptide chain [23]. As a result, the hydrogen bond in the original peptide YR6 may have been destroyed leaving the hydrophilic group exposed, resulting in decreased hydrophobicity of the peptide. The new hexapeptide YAR6 was obtained by replacing the higher HI (3.8) Leu residue with Ala, significantly reducing its hydrophobicity compared with the original hexapeptide YR6; in contrast, the new hexapeptide YGR6 had increased hydrophobicity after substituting the low HI (−1.6) Pro residue with Ala, indicating that the HI of the amino acids had an important impact on the hydrophobicity of the peptide. According to the retention time (Table S3), the order of hydrophobicity of the original and replacement rice peptides is as follows: LR5 > LAR5 > AR5; YGR6 > AGR6 > YR6 > YLR6 > YAR6. Among the YR6 replacement peptides, YGR6 has the strongest hydrophobicity, while AGR6 (AGLYPR) and YLR6 (YGLAPR), which had the same amino acid composition but different sequence orders, had different hydrophobicity. These results suggest that the sequence of amino acids in a peptide affects hydrophobicity.

### 3.2. The O_2_^−^**·** Scavenging Abilities of the Original and Replacement Rice Peptides

**Figure 1:**
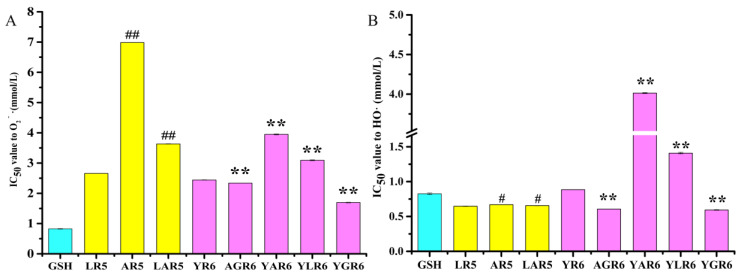
The half maximal inhibitory concentration (IC_50_) value of the original and replacement peptides to the scavenging ability of superoxide anion radicals (O_2_^−^**·**) (**A**) and hydroxyl radicals (HO**·**) (**B**). “#” indicates *p* < 0.05 for significance analysis of pentapeptide, “##” indicates *p* < 0.01 for significance analysis of pentapeptide; “**” indicates *p* < 0.01 for significance analysis of hexapeptide.

The IC_50_ values for the O_2_^−^**·** scavenging ability of the original and replacement rice peptides are shown in Figure 1A. Compared with the original pentapeptide LR5, the IC_50_ values of the replacement pentapeptides AR5 and LAR5 with reduced hydrophobicity increased from 2.6561 ± 0.0031 mmol/L to 6.985 ± 0.0002 mmol/L (*p* < 0.01) and 3.633 ± 0.0071 mmol/L (*p* < 0.01), respectively, indicating that AR5 and LAR5 were less able to scavenge O_2_^−^**·** than the original peptide LR5. Compared with the original hexapeptide YR6, the IC_50_ values of the hexapeptides AGR6 and YGR6 decreased from 2.442 ± 0.0066 mmol/L to 2.339 ± 0.0047 mmol/L (*p* < 0.01) and 1.695 ± 0.0108 mmol/L (*p* < 0.01), respectively, indicating that the scavenging ability of AGR6 and YGR6 for O_2_^−^**·** was stronger than that of the original hexapeptide YR6. Additionally, the IC_50_ values of the hexapeptides YAR6 and YLR6 increased to 3.948 ± 0.0150 mmol/L (*p* < 0.01) and 3.092 ± 0.0140 mmol/L (*p* < 0.01), respectively, suggesting that the O_2_^−^**·** scavenging ability of YAR6 and YLR6 was weaker than that of the original hexapeptide YR6.

### 3.3. The HO**·** Scavenging Abilities of the Original and Replacement Rice Peptides

The IC_50_ values of the original and replacement rice peptides are shown in Figure 1B, which shows that the HO· scavenging ability of the pentapeptides was better than that of the hexapeptides. Compared with the original pentapeptide LR5, the IC_50_ values of the replacement pentapeptides AR5 and LAR5 increased from 0.6476 ± 0.0032 mmol/L to 0.6702 ± 0.0019 mmol/L (*p* < 0.05) and 0.6561 ± 0.0025 mmol/L (*p* < 0.05), respectively, illustrating that pentapeptides AR5 and LAR5 were less able to scavenge HO· than the original pentapeptide LR5. These findings are consistent with the O_2_^−^**·** scavenging ability results that were shown in Figure 1A. Furthermore, compared with the original hexapeptide YR6, the IC_50_ values of the hexapeptides AGR6 and YGR6 decreased from 0.844 ± 0.0020 mmol/L to 0.6065 ± 0.0147 mmol/L (*p* < 0.01) and 0.5929 ± 0.0050 mmol/L (*p* < 0.01), respectively, indicating that the HO·-scavenging capacity of AGR6 and YGR6 was higher than that of the original hexapeptide YR6. The IC_50_ values of the hexapeptides YAR6 and YLR6 increased to 4.013 ± 0.0071 mmol/L (*p* < 0.01) and 1.409 ±0.0120 mmol/L (*p* < 0.01), respectively, indicating that the HO· scavenging ability of YAR6 and YLR6 was lower than that of the original hexapeptide YR6.

### 3.4. Effect of Pre-Incubation with Original and Replacement Rice Peptides on the Anti-Inflammatory Activity in the RAW264.7 Cells

#### 3.4.1. Generation of a Model of Inflammation by Determining the Effects of LPS Concentration on the Proliferation of and Nitric Oxide (NO) Release in the RAW264.7 Cells

**Figure 2:**
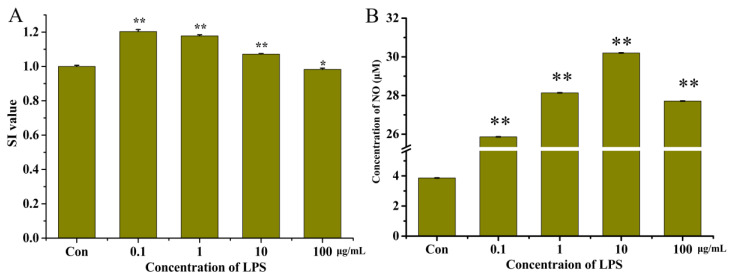
The SI values (**A**) and concentration of NO (**B**) in the RAW264.7 cells after treatment with lipopolysaccharides (LPS) at different concentrations. “*” indicates *p* < 0.05 for significance analysis; “**” indicates *p* < 0.01 for significance analysis.

The effect of different concentrations of LPS on the proliferation of the RAW264.7 cells is shown in Figure 2A. Our results demonstrated that LPS was able to promote the proliferation of the RAW264.7 cells at concentrations of 0.1, 1, and 10 μg/mL. At concentrations of 100 μg/mL (SI = 0.984 ± 0.007), the RAW264.7 cells did not proliferate. Cell proliferation was most similar to the control group when the concentration of LPS was 10 μg/mL (SI = 1.072 ± 0.022).

LPS can induce RAW264.7 cells to produce a large amount of NO, and NO content can be used as an important indicator of the intensity of inflammation in RAW264.7 cells. The effect of different concentrations of LPS on NO released by RAW264.7 cells is shown in Figure 2B. The final LPS concentration was 10 μg/mL (30.12 ± 0.06 μM) at which the concentration of NO was greatest.

To construct a reliable model of inflammation, the concentration of LPS able to stimulate cells to produce the greatest amount of NO with no significant impact on the proliferation of cells should be determined. Therefore, in this study, 10 μg/mL of LPS was chosen as the optimal concentration to construct this model.

#### 3.4.2. Cytotoxicity of the Original and Replacement Rice Peptides in the RAW264.7 Cells

**Figure 3:**
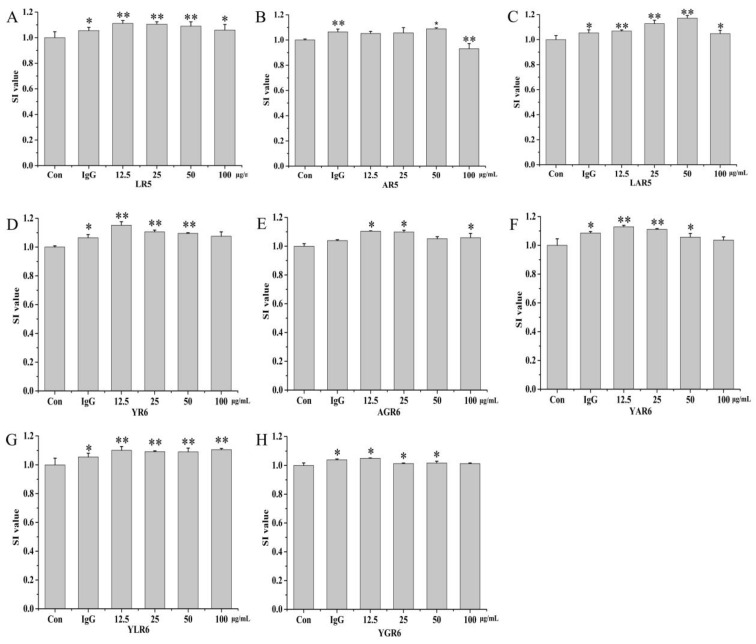
The SI values of the original and replacement peptides. LR5 (**A**); AR5 (**B**); LAR5 (**C**); YR6 (**D**); AGR6 (**E**); YAR6 (**F**); YLR6 (**G**); YGR6 (**H**). “*” indicates *p* < 0.05 for significance analysis; “**” indicates *p* < 0.01 for significance analysis.

To investigate whether the original (LR5 and YR6) and replacement peptides (AR5, LAR5, AGR6, YAR6, YLR6, and YGR6) had inhibitory effects on the proliferation of the RAW264.7 cells or not, cytotoxic effects were investigated at four experimental concentrations of these peptides (12.5, 25, 50, and 100 μg/mL). As shown in Figure 3, the SI values of both the original rice peptides and the replacement peptides were greater than 1.0 across the range of experimental concentrations, indicating that these peptides could promote the proliferation of the RAW264.7 cells and that they showed no cytotoxic effects at these concentrations.

#### 3.4.3. Inhibitory Effects of Peptides on LPS-Induced NO Released by the RAW264.7 Cells

**Figure 4:**
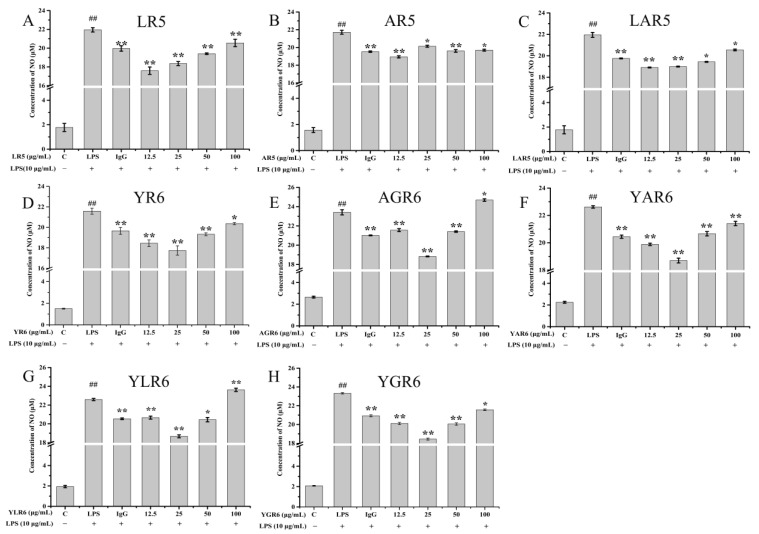
Effect of different concentrations of original and replacement peptides on the amount of NO released from the RAW264.7 mouse macrophage cells. LR5 (**A**); AR5 (**B**); LAR5 (**C**); YR6 (**D**); AGR6 (**E**); YAR6 (**F**); YLR6 (**G**); YGR6 (**H**). “##” indicates *p* < 0.01 for significance analysis of pentapeptide; “*” indicates *p* < 0.05 for significance analysis of peptides; “**” indicates *p* < 0.01 for significance analysis of peptides.

The inhibitory effects of peptides on LPS-induced NO released in the RAW264.7 cells are shown in Figure 4 and Table 1. At a concentration of 12.5 μg/mL, the rate of inhibition of NO release from the RAW264.7 cells by the original peptide LR5 was 19.73%. With the replacement peptides AR5 and LAR5, the inhibition rate was decreased to 12.36% (*p* < 0.01) and 13.84% (*p* < 0.01), respectively. At a concentration of 25.0 μg/mL, the rate of inhibition of NO released from the RAW264.7 cells by the original peptide YR6 was 17.81% (*p* < 0.01). With the replacement peptides YAR6 and YLR6, the inhibition rate decreased to 16.48% (*p* < 0.01) and 17.43% (*p* < 0.01), respectively. The rate of inhibition of NO released from RAW264.7 cells increased to 19.04% (*p* < 0.01) and 22.89% (*p* < 0.01) with the replacement peptides AGR6 and YGR6, which had enhanced hydrophobicity under the same conditions. Most peptides with anti-inflammatory activity contain basic amino acid terminals and hydrophobic amino acids [24]. The results of this study demonstrate that when amino acid replacement with Ala enhances hydrophobicity (as with AGR6 and YGR6), the anti-inflammatory activity of the peptides increased. Conversely, when amino acid replacement with Ala decreases hydrophobicity (as with AR5, LAR5, YAR6, and YLR6), the anti-inflammatory activity of the peptides also decreased, indicating a positive relationship between peptide hydrophobicity and anti-inflammatory activity.

### 3.5. Molecular Docking Analysis of Peptides with the Keap1 Protein

**Figure 5:**
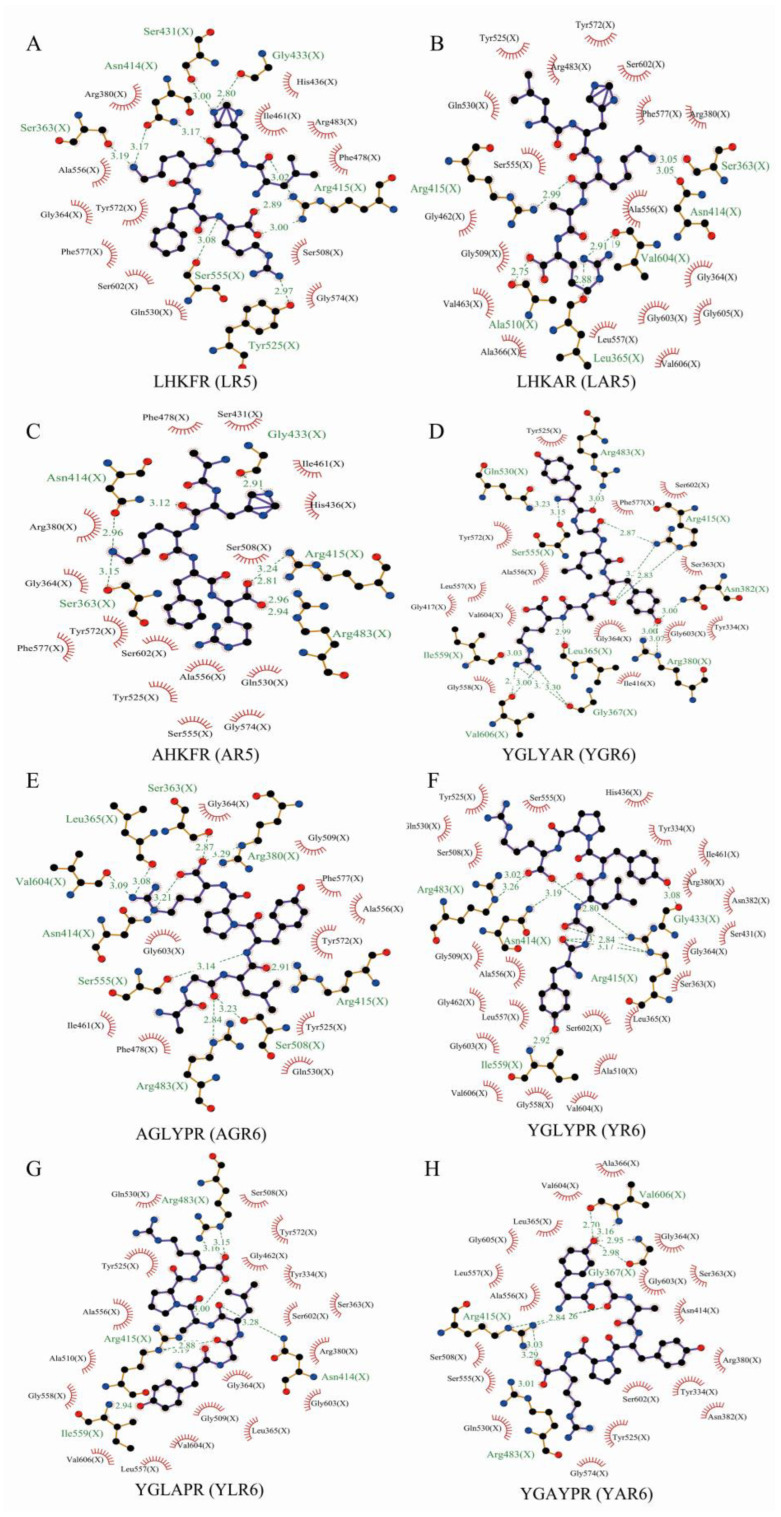
The molecular docking results of different peptides with the Keap1 protein. LR5 (**A**); LAR5 (**B**); AR5 (**C**); YR6 (**D**); YGR6 (**E**); AGR6 (**F**); YLR6 (**G**); YAR6 (**H**).

Molecular docking technology allows us to visualize the details of protein and molecular interactions intuitively at the molecular level. As shown in Figure 5A, the peptide LHKFR (LR5) could interact with serine (Ser) 431, asparagine (Asn) 414, Ser363, Ser555, Tyr525, arginine (Arg) 415, and glycine (Gly) 433 within the Keap1 protein by forming hydrogen bonds. The peptide LHKAR (LAR5) could also interact with Arg415, Ala510, Leu365, Valine (Val) 604, Asn14, and Ser363 within the Keap1 protein by forming hydrogen bonds (Figure 5B). The peptide AHKFR (AR5) formed hydrogen bonds with Arg483, Arg415, Gly433, Asn414, and Ser363 (Figure 5C). The amino acid sequences in the peptides significantly influence the interactions between the peptides and amino acids in the Keap1 protein binding site.

In addition, LAR5 and AR5 formed six and five hydrogen bonds with Keap1, respectively, compared with the original peptide LR5, which formed seven hydrogen bonds with Keap1. The original peptide YR6 formed five hydrogen bonds with Arg483, Asn414, Ile559, Arg415, and Gly433 in Keap1(Figure 5D). However, the number of hydrogen bonds formed by the replacement peptides YGR6 and AGR6, which had increased hydrophobicity, were increased to 10 and 9, respectively; in YGR6, they interacted with Gln530, Ser555, Ile559, Val606, Leu365, Gly367, Arg380, Asn382, Arg415, and Arg 483 (Figure 5E), and in AGR6 they interacted with Ser363, Leu365, Val604, Asn414, Ser555, Arg483, Ser508, Arg415, and Arg380 (Figure 5F). The number of amino acids in the Keap1 protein that interacted with the replacement peptides YLR6 and YAR6, which had reduced hydrophobicity, were both reduced to 4 (Figure 5G,H). These results indicate that the number of hydrogen bonds formed between each peptide and the Keap1 protein was related to the hydrophobicity of the peptide and thus affected their antioxidant performance.

This study has shown that both the original and replacement rice peptides possessed anti-inflammatory and antioxidant activities, and the active sites that determine these characteristics may be the same [25]. Our study of the relationship between peptide structure and activity indicated that peptides containing hydrophobic amino acids (such as Leu, Val, Ala, and Pro) could scavenge free radicals and exert antioxidant activity to a greater extent [25]. Similar conclusions were also reached for the egg white peptide, WNWAD, which effectively eliminated ABTS+**·** free radicals and inhibited the inflammatory factor interleukin-8 (IL-8) and tumor necrosis factor α (TNF-α) in human embryonic kidney (HEK293) cells, demonstrating antioxidant and anti-inflammatory activity [26].

### 3.6. Molecular Dynamics Simulation of Peptides with the Keap1 Protein

**Figure 6:**
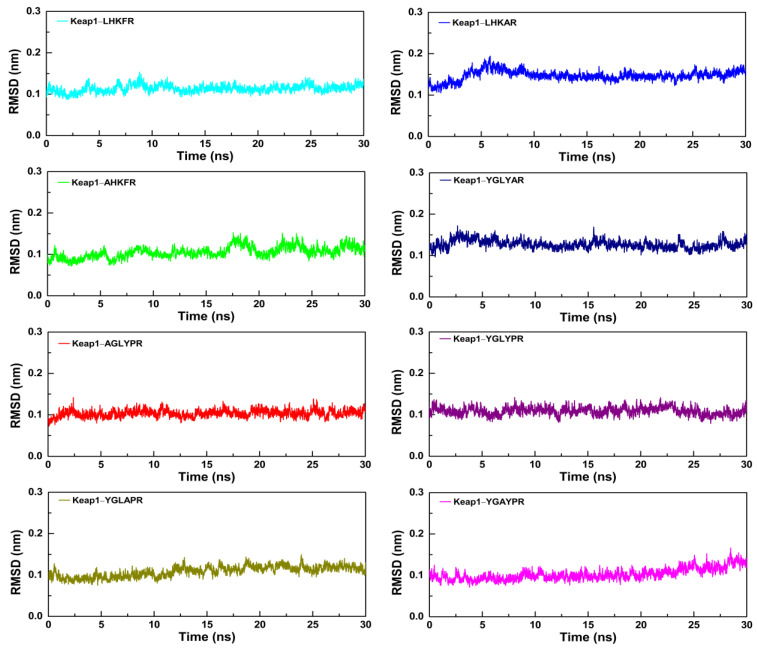
The RMSD values of the different peptides and the Keap1 protein during the molecular dynamics simulation.

**Figure 7:**
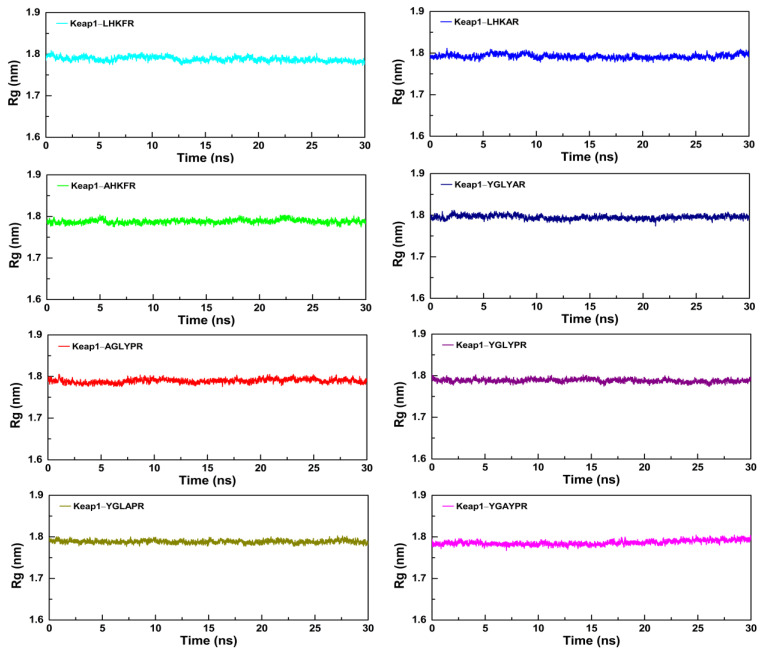
The changes in the Rg values of the different peptides and the Keap1 protein during the molecular dynamics simulation.

**Figure 8:**
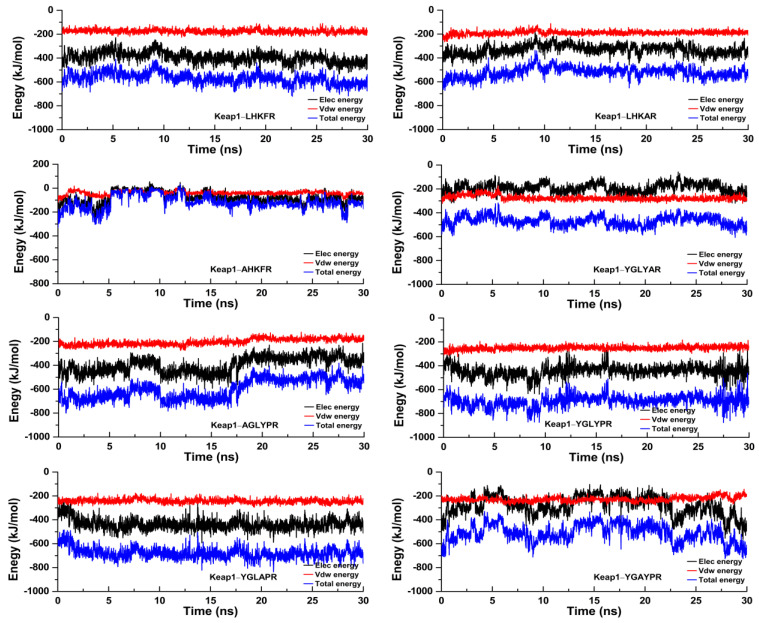
The interactions between the different peptides and the Keap1 protein during the molecular dynamics simulation.

To further study the dynamic conformational changes of peptides with the Keap1 protein, molecular dynamics simulation was used to analyze the root mean square deviation (RMSD), the radius of gyration (Rg), and the interaction energy at 298.15 K for 30 ns. As shown in Figure 6, all the peptide–Keap1 protein complex systems showed certain stability during the simulation process. Among them, the change in the RMSD value of the LR5–Keap1 complex has little fluctuation when compared with AR5–Keap1 and LAR5–Keap1 complexes. All the replacement hexapeptides binding with Keap1 displayed relatively similar RMSD values. Moreover, the values of Rg showed that the bindings of three pentapeptides (LR5, AR5, and LAR5) and five hexapeptides (YR6, AGR6, YAR6, YLR6, and YGR6) to the Keap1 protein did not affect the conformational stability of the protein molecules (Figure 7). We also analyzed the changes in interaction energy including electrostatic interactions (Elec energy) and van der Waals interactions (Vdw energy) between the peptides and Keap1 protein molecule during the simulation process (Figure 8). The results showed that the binding energy of the LR5–Keap1 and LAR5–Keap1 systems were lower than the AR5–Keap1 system, indicating that the interactions of these two systems were stronger. These results were consistent with the above antioxidant and molecular docking results. Furthermore, as shown in Figure 8, electrostatic interactions and van der Waals interactions were important for the binding of the peptides and Keap1, especially electrostatic interactions for the AGR6–Keap1, YR6–Keap1, YLR6–Keap1, and YAR6–Keap1 systems and van der Waals interactions for the YGR6–Keap1 system.

## 4. Conclusions

Compared with the original rice peptides, the peptides AR5, LAR5, YLR6, and YAR6, in which Ala replaced various amino acid residues, had lower hydrophobicity, lower O_2_^−^**·** and HO· scavenging capacity, and lower NO inhibition rates in mouse macrophage cells. Conversely, the replacement peptides AGR6 and YGR6 had enhanced hydrophobicity, greater O_2_^−^**·** and HO· scavenging capacity, and greater NO inhibition rates in mouse macrophage cells. Hydrophobicity and the position of amino acids within the peptides had the same impact on their antioxidant and anti-inflammatory activities. Peptides with strong antioxidant and anti-inflammatory activities may also be hydrophobic and include more basic amino acids. Using amino acid replacement experiments, we found that some replacement peptides have anti-inflammatory and antioxidant activities, which may provide a theoretical basis for the research and development of multifunctional bioactive peptides.

## Figures and Tables

**Table 1:** The inhibitory effect of the original and replacement peptides on LPS-induced NO released by the RAW264.7 cells.

Peptide	NO Inhibition Rate (%)				
	IgG (10 μg/mL)	12.5 μg/mL	25 μg/mL	50 μg/mL	100 μg/mL
LR5	10.19 ± 0.29	19.73 ± 0.39	15.98 ± 0.22	11.05 ± 0.08	8.79 ± 0.39
AR5	9.85 ± 0.08	12.36 ± 0.12	6.76 ± 0.11	9.31 ± 0.15	8.80 ± 0.11
LAR5	9.98 ± 0.04	13.84 ± 0.04	13.51 ± 0.04	11.05 ± 0.04	7.72 ± 0.08
YR6	9.19 ± 0.13	14.51 ± 0.15	17.81 ± 0.34	10.46 ± 0.09	5.65 ± 0.37
AGR6	9.62 ± 0.04	8.04 ± 0.15	19.04 ± 0.04	8.93 ± 0.07	/
YAR6	9.68 ± 0.12	12.28 ± 0.09	16.48 ± 0.18	8.81 ± 016	5.77 ± 0.01
YLR6	9.50 ± 0.09	8.98 ± 0.16	17.43 ± 0.16	9.75 ± 0.23	/
YGR6	10.08 ± 0.11	13.77 ± 0.11	22.89 ± 0.11	13.97 ± 0.13	7.24 ± 0.01

IgG represents immunoglobulin G. “/” indicates there is no inhibition rate.

## Data Availability

Data are available from the first author upon request to cyh@csust.edu.cn or haowu@csust.edu.cn.

## Supplementary Materials

The following supporting information can be downloaded at: https://www.mdpi.com/article/10.3390/nu15102373/s1. Figure S1: The structure of the original and replacement peptides in this study; Figure S2: The RP-HPLC profile of the original peptides and the replacement peptides; Table S1: The detailed amino acid composition information of the original and replacement rice peptides; Table S2: The hydrophilic index of the amino acids; Table S3: The retention time and hydrophobicity results of the original and replacement peptides.
