# Supplementary material for: Alanine Substitution to Determine the Effect of LR5 and YR6 Rice Peptide Structure on Antioxidant and Anti-Inflammatory Activity
Source: Nutrients. 2023 May 18;15(10):2373. doi: 10.3390/nu15102373 (PMC10221837; doi:10.3390/nu15102373)
Supplement: Supplementary file 1 [file nutrients-15-02373-s001.zip › nutrients-2378099-supplementary.pdf]

Supporting material for

Alanine substitution to determine the effect of LR5 and YR6 rice peptide structure on antioxidant and anti-inflammatory activity

Yun-Hui Cheng [1,2], Bu-Qing Liu [2], Bo Cui [1], Li Wen [2], Zhou Xu [2], Mao-Long Chen [2] and Hao Wu [2,*]

[1] School of Food Science and Engineering, Qilu University of Technology (Shandong Academy of Sciences), Jinan 250353, China
[2] School of Food Science and Bioengineering, Changsha University of Science & Technology, Changsha 410114, China
* Correspondence: haowu@csust.edu.cn

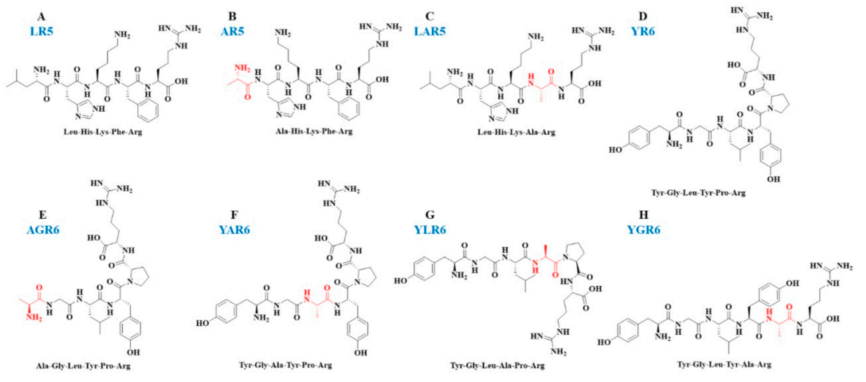

Figure S1 The structure of original and replacement peptides in this study.

Commented [M1]: Please add explanation for subfigures

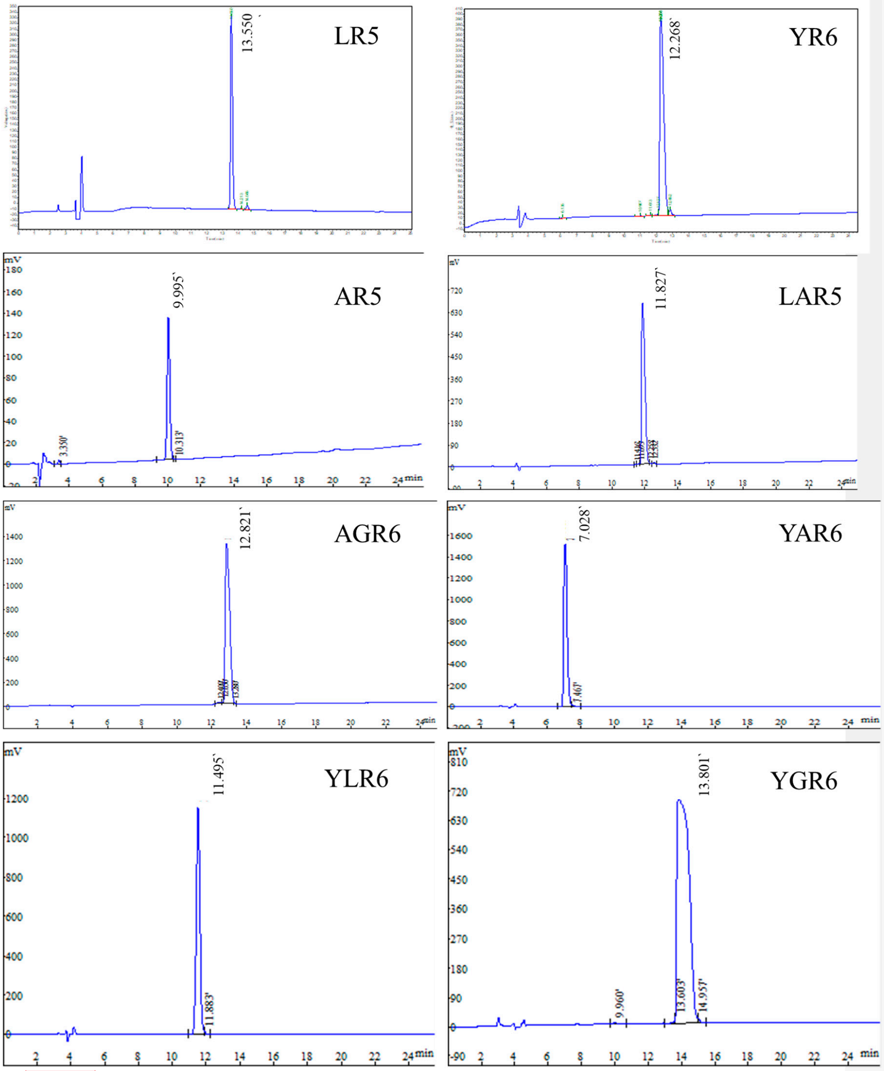

Figure **S2** The RP-HPLC profile of original peptides and replacement peptides

Commented [M2]: The content on the diagram is incomplete and overlapped. Please confirm whether the reading is affected.

Table S1 The detailed amino acid composition information of original and replacement rice peptides.

**Original rice peptides**	**Abbreviated name**	**Amino acid substitution**	**Replacement rice peptides**	**Abbreviated name**
**L**HKFR	LR5	Leu → Ala	**A**HKFR	AR5
LHK**F**R		Phe → Ala	LHK**A**R	LAR5
**Y**GLYPR	YR6	Tyr → Ala	**A**GLYPR	AGR6
YG**L**YPR		Leu → Ala	YG**A**YPR	YAR6
YGL**Y**PR		Tyr → Ala	YGL**A**PR	YLR6
YGLY**P**R		Pro → Ala	YGLY**A**R	YGR6

Commented [M3]: Please confirm if the underline is unnecessary and can be removed. The following highlights are the same

Commented [M4]: Please confirm if the bold is unnecessary and can be removed. The following highlights are the same.

Table S2 The hydrophilic index of amino acids.

**Amino Acid**	**R**	**K**	**N**	**D**	**Q**	**E**	**H**	**P**	**Y**	**W**
**Hydrophilic Index**	-4.5	-3.9	-3.5	-3.5	-3.5	-3.5	-3.2	-1.6	-1.3	-0.9
**Amino Acid**	S	T	G	A	M	C	F	L	V	I
**Hydrophilic Index**	-0.8	-0.7	-0.4	1.8	1.9	2.5	2.8	3.8	4.2	4.5

Table S3 The retention time and hydrophobicity results of original and replacement peptides.

Name	LR5	LAR5	AR5	YR6	AGR6	YAR6	YLR6	YGR6
**Retention Time (mins)**	13.550	11.827	9.995	12.268	12.821	7.028	11.495	13.801
**Hydrophobicity**	LR5 > LAR5 > AR5			YGR6 > AGR6 > YR6 > YLR6 > YAR6				
